# Larvicidal Activity of *Cassia occidentalis* (Linn.) against the Larvae of Bancroftian Filariasis Vector Mosquito *Culex quinquefasciatus*

**DOI:** 10.1155/2014/236838

**Published:** 2014-02-13

**Authors:** Deepak Kumar, Rakesh Chawla, P. Dhamodaram, N. Balakrishnan

**Affiliations:** ^1^Department of Pharmacognosy, Gokula Krishna College of Pharmacy, Nellore District, Sullurpet Andhra Pradesh 524121, India; ^2^Department of Pharma Chemistry, University Institute of Pharmacy, Baba Farid University of Health Sciences, Faridkot, Punjab 151203, India; ^3^Department of Pharmacognosy, Jss College of Pharmacy Rockland, Ooty, Tamil Nadu 643001, India; ^4^National Institute of Communication Diseases Southern India Branch, Coonoor, Tamil Nadu 643101, India

## Abstract

*Background & Objectives*. The plan of this work was to study the larvicidal activity of *Cassia occidentalis* (Linn.) against the larvae of *Culex quinquefasciatus*. These larvae are the most significant vectors. They transmit the parasites and pathogens which cause a deadly disease like filariasis, dengue, yellow fever, malaria, Japanese encephalitis, chikungunya, and so forth, which are considered harmful towards the population in tropic and subtropical regions. *Methods*. The preliminary laboratory trail was undertaken to determine the efficacy of petroleum ether and N-butanol extract of dried whole plant of *Cassia occidentalis* (Linn.) belonging to the family Caesalpiniaceae at various concentrations against the late third instar larvae of *Culex quinquefasciatus* by following the WHO guidelines. *Results*. The results suggest that 100% mortality effect of petroleum ether and N-butanol extract of *Cassia occidentalis* (Linn.) was observed at 200 and 300 ppm (parts per million). The results obviously showed use of plants in insect control as an alternative method for minimizing the noxious effect of some pesticide compounds on the environment. Thus the extract of *Cassia occidentalis* (Linn.) is claimed as more selective and biodegradable agent. *Conclusion*. This study justified that plant *Cassia occidentalis* (Linn.) has a realistic mortality result for larvae of filarial vector. This is safe to individual and communities against mosquitoes. It is a natural weapon for mosquito control.

## 1. Introduction

Mosquito is the most indisputable medicinal significant arthropod vector of diseases. The vector-borne diseases caused by mosquito are one of the major health problems in most of the countries. It is affecting the socioeconomical status of many nations and it is an important pest against human causing allergy too. That includes a local skin reaction [1]. They transmit parasites and pathogens which continue to have disadvantageous impact on human beings [2]. The diseases like filariasis, dengue, yellow fever, malaria, Japanese encephalitis, and chikungunya are some of the deadly diseases spread by mosquitoes. *Culex quinquefasciatus* is an important vector of bancroftian filariasis in tropical and subtropical regions. According to WHO (1984), about 90 million people worldwide were once infected with *Wuchereria bancrofti*, the lymphatic dwelling parasite, and ten time more people are at the risk of being infected. In India alone is 25 million people harbor microfilaria and 19 million people suffer from filarial diseases [3]. Mosquito is frequently found due to poor drainage system especially during rainy seasons, fish pond, and irrigation ditches and rice fields. This provides a better breeding place for mosquitoes [4]. There is provocative interest in research for larvicidal compound from natural sources. Even though chemical vector program has been carried on for long time, these mosquito vectors remain because of repeated use of synthetic products, house hold spray, and insecticides for mosquito control. As a result, the mosquito develops their resistance [5].

Hence, there is a need for developing biologically active natural chemical constituents which act as a larvicidal and promising to reduce the risk to humans and harmful accumulated residues [2, 6]. This has necessitated the need for research and development of environmentally safe, biodegradable, and low cost indigenous method for vector control, which can be used with minimum care by individuals and communities in specific situation [7]. The plant *Cassia occidentalis* (Linn.) is described in Ayurveda and siddha as a potent drug. It is traditionally used for the health disorders like cough, stomachic, constipation, leprosy, and filariasis [8–10]. The extract of whole plant hass proven pharmacologically significant on boils, spasm, hysteria, and whooping cough [11] and also as antioxidant [12], purgative, diuretic, febrifugal, expectorant, stomachic [13], antidiabetic [14], and cytotoxic [15]. The seeds are bitter, sweet, acrid, diuretic, expectorant, purgative, stomachic, febrifuge, and useful in leprosy, ulcer, cough, bronchitis, hiccough, constipation, dyspepsia, and fever. The leaves are bitter, acrid, sweet, thermogenic, depurative, expectorant, and aphrodisiac and are useful in the vitiated condition of *vata* and *kapha* leprosy. The roots are bitter, acrid, thermogenic, diuretic, anti-inflammatory, digestive, stomachic, and tonic used against ring worm, colic flatulence, dyspepsia, epilepsy, and convulsion [16]. Besides the claim of larvicidal property of *Cassia occidentalis* (Linn.), there is no scientific evidence to prove this claim. Hence, the present study has been attempted to make a survey of whole plant extract of *Cassia occidentalis* (Linn.) in a view that the whole plant extract may have most of its biological and biocontrol value when compared to its individual parts, in the context of integrated vector control management. The results of the present study would be useful in promoting research aimed at the development of new agents for mosquito control based on bioactive from of indigenous plant source.

## 2. Materials and Methods

### 2.1. Collection of a Plant Material

The plant was collected during flowering stage in the month of July-August from Nilgris. Then, their identification was established with the aid of an experienced botanist Dr. S. Rajan and compared with herbarium sheets of the authentic sample. Many of defensive components are biodegradable with nonresidual effect on the biological environment; hence, an attempt has been made in present investigation to identify plant with potential to control vector mosquitoes.

### 2.2. Preparation of Extraction

The dried whole plant of *Cassia occidentalis* (Linn.) was powdered and extracted by Soxhlet with petroleum ether and N-butanol. The extracts were concentrated under the rotary vacuum at evaporator until the complete solvents evaporated at (>45°C) to getsemi solid mass of crude extracts [17]. And then use freeze dried (−80°C) to obtain solid residue [18]. These extracts were used for determining the larvicidal activity against mosquito larvae of bancroftian filariasis vector mosquito* Culex quinquefasciatus* [19, 20].

### 2.3. Larvicidal Bioassay

Larvicidal activity was evaluated in accordance with WHO standard with slight modification being done for study [21]. The larvae of *Culex quinquefasciatus* were obtained and reared from the neonatal mosquito in the National Institute of Communicable Disease, Southern India branch field station. This located at Mettupalayam, Coimbatore District, Tamil Nadu. They were maintained at the temperature of 28 ± 2°C and 80 ± 10% RH (relative humidity) under the 12-hour light and dark photoperiod cycle. The larvae were fed dog biscuit and a brewer's yeast powder mixture 3 : 1 ratio is used in the laboratory. After five days, adult male mosquitos were fed a 10% of sucrose solution. The emerging female mosquitoes obtain blood meal from white albino rat for 2-3 h for eggs production [22]. A stock solution was prepared and stored in a refrigerator at 15°C. The 25 healthy late third instar larvae were collected and to composed into two different extracts of Petroleum ether and N-butanol in 500 mL beaker (sterilized glass and plastic beaker) each of which was conducted in triplicate. 25 larvae were collected with a pasture pipette, placed on a filter paper for removal of excess of water and placed in 250 mL dechlorinated tap water containing various concentrations of crude extracts. Three controls in triplicate were set up, with distilled water (250 mL). The beakers were covered with muslin cloth to avoid entry of any foreign material. After exposure the larvae in the extract were observed and outright recorded. In between the experiment, no food was ceded to larvae. At the end of 24 h, the observed mortality (crude mortality) was recorded. There is no sign of any movement even after mild touch with a glass rod [18] and dead larvae are to be counted as described in the WHO technique report series. From this crude mortality, percentage of crude mortality was obtained. Subsequently, control mortality, if any, was recorded and percentage of crude mortality was obtained. The percentage of crude mortality was corrected by Abbot's formula. The corrected probit mortality and expected mortality were also obtained.

### 2.4. Statistical Analysis

The average larval mortality data were subjected to probit analysis for calculating LC_50_ and LC_90_ (lethal concentration) values and their 95% confidence limits were estimated by fitting a probit regression model to the observed relationship between percentage of mortality of larvae and logarithmic concentration of the substance. Separate probit models were fitted for each extract [23]. All of the analysis was carried out using the SPSS (Statistical Package Social Science) software version 13.0.

## 3. Results

The petroleum ether and N-butanol extracts of *Cassia occidentalis* (Linn.) were screened for larvicidal bioassay using water as a vehicle, respectively. The procedure followed for determining larvicidal is the same as described by WHO. The experiments were conducted by using 25 of late third instar larvae for each test solution, keeping a minimum of three replicates and sufficient controls. A total number of 75 larvae were exposed for each concentration for each extract. Five different concentrations of test solution ranging from 40 to 200 ppm were kept for petroleum ether extract and six concentrations of test solution ranging from 50 to 300 ppm were kept for N-butanol extract. The observed and expected mortalities of larvae based on probit regression analysis for different concentrations of *Cassia occidentalis* (Linn.) are shown in Table 1. The estimated LC_50_ and LC_90_ values (95% confidence intervals) for * Cassia occidentalis* (Linn.), Petroleum ether and ethyl N-butanol extracts were 98.4 (89.1–107.2) and 173.8 (160.6–191.5) and 161.6 (152.8–170.5) and 230.0 (217.8–245.5), respectively. The median potency of *Cassia occidentalis* (Linn.) is about 1.6 time less efficacious with petroleum ether and is significantly higher compared to its usage with N-butanol extract. The extracts of these plant have a few important chemical constituents like carbohydrates, glycoside, and tannins [24]. This component has potential larvicidal activity due to individual or synergistic effects on larvae. This needs a further deep investigation to identify the effective constituents for controlling mosquitoes. The results were considered to be statistically significant as given in Table 1.

## 4. Discussion

Globally, there is a prompt awareness going on and always desired to use natural, ecofriendly compounds for larvicidal activity [25]. Mosquito risk has become more acute in recent time and the death of millions of people every year due to mosquito-borne diseases has resulted in the loss of socioeconomic wealth in many countries. The control of mosquito by chemical substance is not safe at present because of insecticide resistance by vectors and environmental imbalance. Application of chemical or synthetic insecticides leads to deleterious effects in the long term, hence it does not provide absolute results. That is why alternative mosquito control method is needed [26]. The extract which is obtained from plant parts like leaves, root, flower, bark, seed, and fruits in their crude extracts has been used as conventional larvicide [25]. The secondary compounds of plants are vast repository of compounds with a wide range of biological activities. Tennyson [17] studied the larvicidal activity of twenty-five plant extracts against the larvae of *Culex quinquefasciatus*. Likewise, Pavela [27] studied the larvicidal activity of thirty-one Euro-Asiatic methanolic extracts against the larvae *Culex quinquefasciatus*. The leaf extract of *Mesua ferra* [28], fruit extract of *Croton caudatus*, flower extract of *Tiliacora acuminate* [29], leaf extract of *Typhonium trilobatum* [30] and flower extract of *Tagetes erecta* [31] were found to cause larval mortality to *Culex quinquefasciatus*. In this study, the petroleum ether and N-butanol extracts of *Cassia occidentalis* (Linn.) whole plant exhibited a dose dependent activity which is similar to other studies which have also reported dose-dependency of plant against mosquito larvae [32–34]. The mechanism of action exhibited by *Cassia occidentalis* (Linn.) whole plant may therefore possibly be due to its toxic effects on the larvae. This is supported by a previous study which reported that an ethanol extract of *P. Pinnata* exhibited toxic effects in the Swiss albino mice [35]. Other studies also showed clearly that the chloroform and methanolic extracts of *Tagetes minuta* L. have a pronounced larvicidal effect on mosquito Anopheles stephensi which is due to the presence of the phytochemicals such as flavonoids, phenols, terpenoids, and saponins. Thus, the above statement may potentiate that the shown larvicidal activity of *Cassia occidentalis* (Linn.) whole plant may also be due to presence of flavonoids, phenols, glycosides, and tannins.

Hence, from the present study, the authors concluded that the petroleum ether and N-butanol extracts of *Cassia occidentalis* (Linn.) whole plant extract have larvicidal activities.

## 5. Conclusion

On the basis of the above results, we can conclude that *Cassia occidentalis* (Linn.) has a paramount larvicidal importance. The synthetic chemical can be obtained easily at a very low cost. But the use of the plants for larvae control offers a safer alternative too. Moreover, these results could be useful in the search for newer compounds. These extracts are inexpensive, easy to handle, and safer products for the control of mosquito larvae.

## Figures and Tables

**Table 1:** Observed and expected mortality of *Culex quinquefasciatus* larvae exposed to *Cassia occidentalis* with petroleum ether and N-butanol extracts. Expected mortality is based on probit regression analysis.

Conc. (*μ*g/mL)	Number of larvae		Mortality (%)		Expected mortality			LC_50_ (95% CI)	LC_90_ (95% CI)	Probit (mortality) = *a* + *b* × conc.	*χ* ^2^ D.F. *P* value
	Exposed	Dead	Crude	Corrected	Probit	Dead	%				
*Cassia occidentalis* with petroleum ether											
40	75	15	20	20	−1.00	12.0	16.0	98.4 (89.1–107.2)	173.8 (160.6–191.5)	−1.6756 + 0.0170 × conc.	*χ* ^2^ = 6.20 D.F. = 3 *P* = 0.1
80	75	28	37.4	37.4	−0.32	28.2	37.6				
120	75	43	57.4	57.4	0.36	48.2	64.2				
160	75	62	82.7	82.7	1.04	63.9	85.2				
200	75	75	100	100	1.72	71.8	95.8				

*Cassia occidentalis* with N-butanol											
50	75	3	4	4	−2.09	1.4	1.8	161.6 (152.8–170.5)	230.0 (217.8–245.5)	−3.0289 + 0.0187 × conc.	*χ* ^2^ = 3.24 D.F. = 4 *P* = 0.5
100	75	7	9.4	9.4	−1.16	9.2	12.3				
150	75	31	42.7	42.7	−0.22	30.9	41.1				
200	75	56	74.7	74.7	0.71	57.1	76.1				
250	75	72	96	96	1.65	71.3	95.0				
300	75	75	100	100	2.58	74.6	99.5				

D.F.: degrees of freedom.
